# Increased intraocular inflammation in retinal vein occlusion is independent of circulating immune mediators and is involved in retinal oedema

**DOI:** 10.3389/fnins.2023.1186025

**Published:** 2023-07-24

**Authors:** Yufan Zhou, Jinyan Qi, Hengwei Liu, Shengnan Liang, Tingting Guo, Juan Chen, Wei Pan, Huanhuan Tan, Jie Wang, Heping Xu, Zhongping Chen

**Affiliations:** ^1^Changsha Aier Eye Hospital, Changsha, China; ^2^Aier School of Ophthalmology, Central South University, Changsha, China; ^3^Aier Institute of Optometry and Vision Science, Changsha, China; ^4^The First Clinical Medical College of Jinan University, Guangzhou, China; ^5^Wellcome-Wolfson Institute for Experimental Medicine, School of Medicine, Dentistry and Biomedical Sciences, Queen’s University Belfast, Belfast, United Kingdom; ^6^School of Stomatology and Ophthalmology, Xianning Medical College, Hubei University of Science and Technology, Xianning, China

**Keywords:** aqueous humor, inflammatory factors, plasma, optical coherence tomographic angiography, VEGF, PI3 K signaling pathway, JAK-STAT signaling pathway

## Abstract

We aim to understand the link between systemic and intraocular levels of inflammatory mediators in treatment-naïve retinal vein occlusion (RVO) patients, and the relationship between inflammatory mediators and retinal pathologies. Twenty inflammatory mediators were measured in this study, including IL-17E, Flt-3 L, IL-3, IL-8, IL-33, MIP-3β, MIP-1α, GRO β, PD-L1, CD40L, IFN-β, G-CSF, Granzyme B, TRAIL, EGF, PDGF-AA, PDGF-AB/BB, TGF-α, VEGF, and FGFβ. RVO patients had significantly higher levels of Flt-3 L, IL-8, MIP-3β, GROβ, and VEGF, but lower levels of EGF in the aqueous humor than cataract controls. The levels of Flt-3 L, IL-3, IL-33, MIP-1α, PD-L1, CD40 L, G-CSF, TRAIL, PDGF-AB/BB, TGF-α, and VEGF were significantly higher in CRVO than in BRVO. KEGG pathway enrichment revealed that these mediators affected the PI3K-Akt, Ras, MAPK, and Jak/STAT signaling pathways. Protein–Protein Interaction (PPI) analysis showed that VEGF is the upstream cytokine that influences IL-8, G-CSF, and IL-33 in RVO. In the plasma, the level of GROβ was lower in RVO than in controls and no alterations were observed in other mediators. Retinal thickness [including central retinal thickness (CRT) and inner limiting membrane to inner plexiform layer (ILM-IPL)] positively correlated with the intraocular levels of Flt-3 L, IL-33, GROβ, PD-L1, G-CSF, and TGF-α. The size of the foveal avascular zone positively correlated with systemic factors, including the plasma levels of IL-17E, IL-33, INF-β, GROβ, Granzyme B, and FGFβ and circulating high/low-density lipids and total cholesterols. Our results suggest that intraocular inflammation in RVO is driven primarily by local factors but not circulating immune mediators. Intraocular inflammation may promote macular oedema through the PI3K-Akt, Ras, MAPK, and Jak/STAT signaling pathways in RVO. Systemic factors, including cytokines and lipid levels may be involved in retinal microvascular remodeling.

## 1. Introduction

Retinal vein occlusion (RVO) is an obstruction of the retinal venous system that can occur in a branch of the retinal vein (branch retinal vein occlusion, BRVO) or the central retinal vein (central retinal vein occlusion, CRVO). The pathogenesis of the disease remains elusive. The risk factors of RVO include both local (e.g., glaucoma, short axial distance) and systemic (e.g., hypertension, arteriosclerosis, hypercholesterolemia, diabetes mellitus, systemic vascular disease or inflammation, inherited thrombophilia, increased coagulability, age, obesity, smoking, etc.; Marcinkowska et al., 2022). RVO causes retinal hemorrhage, ischemia, macular oedema, and neovascularization leading to visual impairment (Jung et al., 2014; Zeng et al., 2019). The mainstream therapies of RVO include intraocular injection of vascular endothelial cell growth factor (VEGF) inhibitors or dexamethasone, laser photocoagulation, and surgery (Soliman et al., 2022). Despite the successful outcomes of intraocular injection of anti-VEGF agents, up to 77.5% of RVO patients continue to suffer from refractory or recurring edema (Karagiannis et al., 2011). There is an urgent need to understand the pathogenesis of the disease and the mechanism of retinal damage, especially persistent macular oedema.

Blood vessel occlusion is caused by abnormal thrombosis, which can arise from abnormal activation of the platelets, circulating immune cells, and vascular endothelial cells (Rayes et al., 2020; Pilard et al., 2022). Previous studies have shown platelets response to thrombin and collagen (Leoncini et al., 2007) is increased in RVO patients. Higher circulating neutrophil counts and monocyte/high-density lipoprotein ratio have been reported in RVO patients (Duru et al., 2021; Pan et al., 2022). Patients with ocular tuberculosis have been reported to present as BRVO with (Yuksel and Ozdek, 2013) or without (O'Hearn et al., 2007) retinal vasculitis or uveitis, suggesting the involvement of local inflammation in RVO. Furthermore, higher levels of inflammatory mediators such as IL-6 (Yong et al., 2021), IL-8 (Yang et al., 2021), MCP-1 (Wei et al., 2020), placental growth factor (PIGF; Noma et al., 2014), platelet-derived growth factor (PDGF; Lee et al., 2012), and adhesion molecule ICAM-1 (Yi et al., 2020) have been reported in RVO patients. Inflammation and hypoxia are believed to play a critical role in the pathogenesis of RVO (Noma et al., 2020). How the systemic and local factors work together to lead to RVO remains unknown.

In this study, we measured the levels of 20 inflammatory mediators, including eight inflammatory cytokines (IL-17E, Flt-3 L, IL-3, IL-8, IL-33, MIP-3β, MIP-1α, and GRO β), six immunomodulatory cytokines (PD-L1, CD40L, IFN-β, G-CSF, Granzyme B, and TRAIL), and six growth factors involved in extracellular matrix remodeling and angiogenesis (EGF, PDGF-AA, PDGF-AB/BB, TGF-α, VEGF, and FGFβ) in both the plasma and the aqueous humor of RVO. We further investigated the relationship between the inflammatory mediators and clinical presentations (e.g., visual acuity, retinal thickness, and vascular density etc.) of RVO. Surprisingly, we found that increased intraocular inflammation in RVO is independent of systemic immune mediators.

## 2. Materials and methods

### 2.1. Patients

This prospective observational case–control study was performed under the Declaration of Helsinki. Informed consent was obtained from each participant and the Institutional Review Board (IRB) at the Changsha Aier Eye Hospital approved the study [Ethical approval number: (2020) KYPJ005]. Twenty RVO (six with CRVO and 14 with BRVO) treatment-naive patients were recruited from January 2021 to December 2021 in Changsha Aier Eye Hospital. Inclusion criteria were (1) RVO confirmed by fundus stereoscopy, optical coherence tomography angiography (OCTA), and fluorescein fundus angiography (FFA); (2) RVO with visual impairment who require medical attention [e.g., intravitreal anti-VEGF (ranibizumab) or dexamethasone (ozurdex) treatment]. Twenty patients with senile cataracts undergoing cataract surgery were used as controls. The inclusion criteria of the control group were elderly cataract patients who needed phacoemulsification. The exclusion criteria for all participants were (1) other retinal pathologies (e.g., glaucoma, diabetic retinopathy, and retinal neovascularization by other causes); (2) the existence or history of intraocular or systemic inflammatory diseases (e.g., uveitis, active pneumonitis); (3) the presence or history of cancer (e.g., breast cancer, colon cancer), autoimmune diseases (e.g., systemic lupus erythematosus), ocular trauma, severe liver, and kidney dysfunction; (4) history of intraocular surgery (e.g., vitrectomy，anti-VEGF, intraocular steroids) or laser treatment within 6 months.

### 2.2. Data collection and masking

All clinical examinations were conducted by two consultant ophthalmologists. Retinal images (FFA, OCT, and OCTA) were examined by two experienced retinal specialists. Researchers who carried out laboratory experiments and analysis were masked to the origin of the clinical samples to be analyzed.

### 2.3. Ocular examination

All RVO patients underwent a complete ocular examination including logMAR best corrected visual acuity (BCVA), slit lamp examination, intraocular pressure measurement, B-scan ocular ultrasound and fundus stereoscopic biomicroscopy, FFA (Spectralis™ HRA, Heidelberg Engineering, Heidelberg, Germany), and OCTA (RTVue-XR Avanti, Optovue, Fremont, CA, United States). OCTA images were used to collect the below parameters: CRT, foveal avascular zone (FAZ), perimeter (PERIM) of the fovea, ILM-IPL thickness, superficial vessel density (SVD), and deep vessel density (DVD).

All cataract patients underwent a comprehensive ophthalmic examination before surgery, including Log MAR BCVA, slit-lamp examination, intraocular pressure measurement, fundus stereoscopic biomicroscopy, B-scan ocular ultrasound, and optical coherence tomography (OCT; CIRRUS ™HD-OCT 5000, Carl Zeiss Meditec, Dublin, CA, United States).

### 2.4. Blood test

The following parameters were collected from routine blood tests: glucose (GLU), triglyceride (TG), total cholesterol (TC), high-density lipoprotein cholesterol (HDLC), low-density lipoprotein cholesterol (LDLC), prothrombin time (PT), prothrombin time-international normalized ratio (PTINR), thrombin time (TT), activated partial thromboplastin time (APTT), and fibrinogen (FIB).

### 2.5. Demographic information

The following information was extracted from the medical records of each patient: age, gender, body mass index (BMI), the use of other medications (e.g., to control hypertension, diabetes, aspirin, and hormone supplements), smoking history, other eye diseases, history of systemic disease (e.g., hypertension, diabetes), history of allergy disease, autoimmune diseases, surgical history, history of drug allergy, and history of vaccinations.

### 2.6. Sample collection

The aqueous humor and plasma samples were collected as described previously (Luo et al., 2021). Briefly, in RVO patients, 60 μL of aqueous humor was extracted before intravitreal injection of ranibizumab or dexamethasone to relieve intraocular pressure. In cataract patients, 50 μL of aqueous humor was collected at the beginning of cataract surgery procedure. All procedures were conducted in an ophthalmology surgical operating room.

Fasting blood samples (5 mL) were loaded into a purple sterile vacuum blood collection tube with EDTAK2 anticoagulant (Huabo Technology, Haze, China) and were reversed several times immediately. Approximately, 2 mL of plasma samples were collected after centrifugation (3,000 r/min, 10 min at room temperature). Plasma and aqueous humor samples were kept in sterile Eppendorf and stored at −80°C until laboratory measurements.

### 2.7. Laboratory measurement

The levels of IL-17E, Flt-3 L, IL-3, IL-8, IL-33, MIP-3β, MIP-1α, GRO β, PD-L1, CD40 L, IFN-β, G-CSF, Granzyme B, TRAIL, EGF, PDGF-AA, PDGF-AB/BB, TGF-α, VEGF, and FGFβ in aqueous humor and plasma samples were measured using the Luminex® × MAP® technology following manufacturer’s instructions (Luminex × Map Technology, Bio-Rad). A total of 25 μL of aqueous humor (1:2 dilution) and plasma (undiluted) from each sample were used in the study, respectively. The fluorescent intensity was measured using a plate reader with the Luminex® × PONENT® acquisition software (MAGPIX®). The concentration of target molecules was calculated using the MILLIPLEX® Analyst 5.1.

### 2.8. Bioinformatics analysis

Kyoto Encyclopedia of Genes and Genomes (KEGG) enrichment pathway analysis of differentially expressed proteins (DEPs) was performed using the online platform OmicShare^1^ with adjusted *p* < 0.05. A protein–protein interaction (PPI) network of DEPs was searched in the STRING database with a confidence score > 0.4. The top 10 DEPs with the highest connection degree were calculated by the Cytohubba plugin and visualized in Cytoscape (Version3.9.1, Oracle, Redwood City, CA, United States).

### 2.9. Statistical analysis

Data were analyzed using the SPSS 20.0 software and plots were generated using GraphPad Prism v8.0. The Kolmogorov–Smirnov test was used to evaluate the data distribution. The non-normally distributed data were log-transformed into a normal distribution. The continuous variables were presented as mean ± SD and compared by the Student’s *t*-test and One-way ANOVA. The chi-square test was used to compare categorical variables. Multivariable linear regression was used to check confounding factors when comparing the variables in different groups. The correlation between aqueous humor/plasma inflammatory factors levels and clinical parameters was assessed using the Pearson correlation method. *p* < 0.05 was considered statistically significant.

## 3. Results

### 3.1. Laboratory measurement rate

Cytokine levels were measured in 32 aqueous humor samples (13 controls and 19 RVO) and 40 plasma samples (20 controls and 20 RVO). All cytokines were within the measurement range. One sample in the cataract group was excluded from the analysis because 50% of the measurements were extremely high and the participant was later confirmed to suffer from rheumatoid arthritis.

### 3.2. Clinical characteristics

There was no significant difference in gender distribution, history of hypertension or diabetes, history of smoking, BMI, and BCVA between RVO patients and controls (Table 1). However, the average age of RVO, subgroup-BRVO patients was significantly younger than that of controls (Table 1). As expected, the CRT of RVO patients (including subgroup CRVO and BRVO) were significantly higher than controls (Table 1). There was no significant difference in age, RVO duration，CRT, FAZ, PERIM, ILM-IPL, SVD, and DVD between BRVO and CRVO (Table 1).

**Table 1:** Demographic and clinical characteristics of study participants.

			Subgroups of RVO		
Clinical variables	Control	RVO	CRVO	BRVO	*p* values
	*N* = 19	*N* = 20	*N* = 6	*N* = 14	BRVO vs. CRVO
Age (mean ± SD) years	66.63 ± 5.29	**59.55 ± 11.44** ^*, a^	60.17 ± 11.04	**59.29 ± 12.00** ^*, c^	1.000
Male (%)^b^	52.6	60.00	16.67	50.00	0.163
Hypertension (%)^b^	42.10	55.00	16.66	71.43	0.111
Diabetes (%)^b^	10.50	10.00	16.66	7.14	0.515
Smoking (%)^b^	26.30	15.00	16.66	14.29	0.891
BMI (mean ± SD) kg/m^2^	22.13 ± 3.58	24.83 ± 3.21	23.7 ± 2.62	25.32 ± 3.40	1.00
BCVA (mean ± SD)	0.93 ± 0.85	0.92 ± 0.28	0.97 ± 0.24	0.90 ± 0.31	1.000
RVO duration (≤ 1-month) (%)^b^	\	50	50	50	1.000
CRT (mean ± SD) μm	222.22 ± 25.91	**588.85 ± 221.26** ^**, a^	**660.50 ± 215.33** ^**, c^	**558.14 ± 224.35** ^**, c^	0.56
FAZ (mean ± SD) mm^2^	\	0.24 ± 0.14	0.23 ± 0.13	0.24 ± 0.15	0.966
PERIM (mean ± SD) mm	\	1.93 ± 0.57	2.05 ± 0.58	1.89 ± 0.58	0.582
ILM-IPL (mean ± SD)	\	125.82 ± 96.88	169.11 ± 121.79	107.26 ± 82.33	0.199
SVD (mean ± SD)	\	44.75 ± 3.77	44.57 ± 3.21	44.82 ± 4.09	0.894
DVD (mean ± SD)	\	44.63 ± 3.23	45.91 ± 2.68	44.08 ± 3.37	0.255

^a^Independent sample *t*-test, between controls and RVO patients; ^b^Chi-square test; ^c^One-way ANOVA test analyzed controls, CRVO, and BRVO patients. ^*^p < 0.05; ^**^p < 0.01. Bold indicating *p* value was statistically significant. RVO, retinal vein occlusion; BRVO, branch retinal vein occlusion; CRVO, central retinal vein occlusion; BMI, body mass index; BCVA, best corrected visual acuity (expressed as LogMAR); CRT, central retinal thickness; FAZ, foveal avascular zone; PERIM, perimeter of fovea; ILM-IPL, inner limiting membrane-inner plexiform layer; SVD, superficial vessel density; and DVD, deep vessel density.

### 3.3. Inflammatory mediators in RVO patients and controls

In the aqueous humor, the levels of Flt-3 L, IL-8, MIP-3β, GROβ, PDGF-AA, and VEGF in the RVO patients were significantly higher while the level of EGF was significantly lower than in controls (Table 2). After adjusting for age, except for PDGF-AA, the differences remained (Table 2). Further subgroup analyses showed that the levels of Flt-3 L, IL-8, IL-33, MIP-1α, GROβ, G-CSF, PDGF-AA, PDGF-AB/BB, TGF-α, and VEGF in CRVO patients were significantly higher than those in controls after adjustment for age (Table 2). The levels of Flt-3 L, IL-8, MIP-3β, and GROβ in BRVO patients were also significantly higher than in controls. However, the levels of PD-L1, CD40L, EGF, IL-3, and TRAIL in BRVO were significantly lower than in the controls (Table 2). Furthermore, patients with CRVO had significantly higher levels of Flt-3 L, IL-3, IL-33, MIP-1α, PD-L1, CD40L, G-CSF, TRAIL, PDGF-AB/BB, TGF-α, and VEGF than patients with BRVO (Table 2).

**Table 2:** Aqueous humor levels of inflammatory factors in RVO patients and controls.

			Subgroups of RVO		
Variables	Control	RVO	CRVO	BRVO	*p* values
	(*n* = 13, pg/mL)	(*n* = 19, pg/mL)	(*n* = 5, pg/mL)	(*n* = 14, pg/mL)	BRVO vs. CRVO
IL-17E	21.15 ± 1.76	21.30 ± 2.60	22.14 ± 3.01	21.00 ± 2.49	0.445
Flt-3 L	30.53 ± 1.99	**40.95 ± 11.63** ^**, a^	**51.00 ± 11.11** ^**, c^	**37.36 ± 9.83** ^*, c^	**0.022** ^b^
IL-3	25.91 ± 2.79	24.26 ± 2.29	25.98 ± 3.46	**23.64 ± 1.41** ^*, c^	**0.047** ^b^
IL-8	13.86 ± 7.18	**101.40 ± 82.64** ^**, a^	**177.04 ± 103.40** ^**, c^	**74.38 ± 56.38** ^**, c^	0.063
IL-33	17.40 ± 1.31	17.96 ± 1.28	**19.22 ± 1.19** ^**, c^	17.50 ± 0.99	**0.006** ^b^
MIP-3β	6.30 ± 0.79	**13.19 ± 8.78** ^**, a^	9.6 ± 2.31	**14.47 ± 9.92** ^*, c^	0.388
MIP-1α	13.35 ± 2.04	13.29 ± 1.51	**14.94 ± 1.46** ^*, c^	12.69 ± 1.03	**0.002** ^b^
GRO β	14.70 ± 1.82	**21.64 ± 7.12** ^**, a^	**26.73 ± 7.46** ^**, c^	**19.83 ± 6.29** ^*, c^	0.065
PD-L1	109.26 ± 8.61	104.12 ± 12.76	119.10 ± 14.53	**98.78 ± 6.58** ^**, c^	**0.001** ^b^
CD40 L	1440.41 ± 162.50	1336.93 ± 129.86	1437.08 ± 137.22	**1301.17 ± 111.01** ^**, c^	**0.047** ^b^
IFN-β	3.87 ± 0.44	3.95 ± 0.40	3.77 ± 0.37	4.02 ± 0.41	0.254
G-CSF	10.11 ± 1.25	61.11 ± 151.63	**201.45 ± 264.60** ^**, c^	10.99 ± 3.23	**0.008** ^b^
Granzyme B	8.98 ± 2.12	9.60 ± 3.23	11.86 ± 5.46	8.80 ± 1.62	0.125
TRAIL	26.62 ± 1.53	26.28 ± 1.57	27.76 ± 1.81	**25.75 ± 1.21** ^*, c^	**0.01** ^b^
EGF	9.72 ± 1.47	**8.57 ± 1.95** ^*, a^	9.39 ± 1.24	**8.27 ± 2.11** ^*, c^	0.173
PDGF-AA	219.25 ± 36.00	292.94 ± 101.29	**371.61 ± 127.45** ^**, c^	264.84 ± 77.30	0.083
PDGF-AB/BB	3.85 ± 0.19	3.86 ± 0.26	**4.07 ± 0.29** ^*, c^	3.78 ± 0.21	**0.032** ^b^
TGF-α	9.84 ± 0.67	9.99 ± 0.79	**11.04 ± 0.53** ^**, c^	9.62 ± 0.47	**0.000** ^b^
VEGF	278.84 ± 123.40	**829.49 ± 892.55** ^*, a^	**1599.37 ± 1118.44** ^**, c^	554.53 ± 639.36	**0.014** ^b^
FGF β	12.80 ± 2.00	12.84 ± 2.56	13.14 ± 1.56	12.73 ± 2.88	0.641

^a^Multivariable linear regression analysis of inflammatory factors between controls and RVO patients after adjusting for age. ^b^Independent sample *t*-test analysis between BRVO and CRVO patients; ^c^Multivariable linear regression analysis of inflammatory factors between controls and CRVO, BRVO patients after adjusting for age. ^*^p < 0.05; ^**^p < 0.01. Bold indicating p value was statistically significant. RVO, retinal vein occlusion; BRVO, branch retinal vein occlusion; and CRVO, central retinal vein occlusion.

In the plasma, the levels of GROβ, EGF in the RVO patients were significantly lower than in controls. After adjusting for age, the difference in GROβ remained (Table 3). Further subgroup analysis showed that the level of GROβ in both CRVO and BRVO was significantly lower than that in controls (Table 3). The level of MIP-3β in BRVO patients was significantly higher than in controls after adjustment for age (Table 3). There was no significant difference in any of the variables between CRVO and BRVO patients (Table 3). We further found that the plasma levels of IL-17E, IL-8, IL-33, MIP-1α, TGF-α, and FGFβ in RVO positively correlated with disease duration, and patients with >1-month disease duration had significantly higher levels of these cytokines than patients with ≤1 month-disease duration (Supplementary Table S1). However, there was no significant difference in aqueous inflammatory mediators between disease duration >1-month and disease duration ≤1-month (Supplementary Table S1).

**Table 3:** Plasma levels of inflammatory mediators in RVO patients and controls.

			Subgroups of RVO		
Variables	Control	RVO	CRVO	BRVO	*p* values
	(*n* = 19, pg/mL)	(*n* = 20, pg/mL)	(*n* = 6, pg/mL)	(*n* = 14, pg/mL)	BRVO vs. CRVO
IL-17E	6.22 ± 3.47	7.80 ± 6.96	7.63 ± 7.77	7.88 ± 6.89	0.81
Flt-3 L	66.15 ± 19.28	64.50 ± 28.82	63.23 ± 28.88	65.04 ± 29.86	0.987
IL-3	4.19 ± 3.90	5.35 ± 11.03	4.86 ± 3.90	5.55 ± 13.11	0.231
IL-8	2.87 ± 1.36	4.62 ± 8.21	1.76 ± 0.97	5.84 ± 9.63	0.394
IL-33	10.64 ± 4.51	9.75 ± 9.09	9.84 ± 7.98	9.71 ± 9.81	0.848
MIP-3β	61.91 ± 18.64	79.86 ± 34.22	68.44 ± 18.73	**84.76 ± 38.61** ^*, c^	0.413
MIP-α	20.12 ± 15.00	15.96 ± 10.40	15.04 ± 8.89	16.36 ± 11.27	0.992
GRO β	316.98 ± 170.94	**82.37 ± 102.26** ^**, a^	**71.67 ± 69.24** ^**, c^	**86.96 ± 115.61** ^**, c^	0.872
PD-L1	179.35 ± 433.37	118.37 ± 120.52	197.58 ± 147.27	84.42 ± 93.55	0.057
CD40 L	365.81 ± 195.71	562.85 ± 1179.37	483.26 ± 441.56	596.96 ± 1397.75	0.368
IFN-β	3.31 ± 4.18	3.42 ± 3.97	3.73 ± 5.51	3.28 ± 3.36	0.837
G-CSF	13.71 ± 7.74	11.89 ± 11.23	13.05 ± 13.56	11.39 ± 10.62	0.768
Granzyme B	3.40 ± 3.59	4.31 ± 8.90	5.08 ± 5.83	3.98 ± 10.11	0.225
TRAIL	43.28 ± 19.78	53.73 ± 32.19	60.96 ± 23.66	50.63 ± 35.56	0.301
EGF	15.24 ± 25.67	11.48 ± 22.61	11.22 ± 17.35	11.59 ± 25.12	0.548
PDGF-AA	1838.86 ± 1119.14	1690.93 ± 2137.33	1580.28 ± 2042.11	1738.36 ± 2250.46	0.961
PDGF-AB/BB	356.67 ± 291.63	299.83 ± 373.05	371.66 ± 516.67	269.05 ± 311.96	0.616
TGF-α	9.48 ± 6.44	9.10 ± 9.50	8.37 ± 8.91	9.41 ± 10.06	0.774
VEGF	56.57 ± 19.56	63.40 ± 53.77	78.57 ± 85.06	56.90 ± 35.93	0.601
FGF β	11.74 ± 6.34	11.29 ± 13.72	10.46 ± 11.55	11.65 ± 14.94	0.928

^a^Multivariable linear regression analysis of inflammatory factors between controls and RVO patients after adjusting for age. ^b^Independent sample *t*-test analysis between BRVO and CRVO patients; ^c^Multivariable linear regression analysis of inflammatory factors between controls and CRVO, BRVO patients after adjusting for age. ^*^p < 0.05; ^**^p < 0.01. Bold indicating *p* value was statistically significant. RVO, retinal vein occlusion; BRVO, branch retinal vein occlusion; and CRVO, central retinal vein occlusion.

### 3.4. Correlation between inflammatory mediators and OCTA parameters in RVO patients

Pearson correlation analysis showed aqueous humor levels of GROβ and Granzyme B positively correlated with the CRT. The aqueous levels of Flt-3 L, IL-33, GROβ, PD-L1, G-CSF, and TGF-α positively correlated with the ILM-IPL thickness. G-CSF positively correlated and MIP-3β negatively correlated with DVD (Table 4). No correlation was observed between the aqueous humor levels of inflammatory factors and visual acuity, PERIM, FAZ, and SVD (Table 4). Our results suggest that intraocular levels of Flt-3 L, IL-33, GROβ, PD-L1, G-CSF, TGF-α, and MIP-3β may contribute to vascular leakage and retinal deep layer vascular degeneration in RVO.

**Table 4:** Correlation between inflammatory factors and BCVA and OCTA parameters in aqueous humor RVO patients (*n* = 19).

Variables	Log MAR	CRT	FAZ	PERIM	ILM-IPL	SVD	DVD
	*r*	*r*	*r*	*r*	*r*	*r*	*r*
IL-17E	0.12	−0.12	0.45	0.20	−0.11	−0.02	0.15
Flt-3 L	−0.02	0.34	−0.32	0.04	**0.57** ^**^	0.19	0.12
IL-3	−0.25	0.42	0.24	0.11	0.39	−0.13	0.14
IL-8	−0.06	0.27	−0.44	−0.04	0.41	0.01	0.12
IL-33	0.04	0.36	0.05	0.12	**0.53** ^*^	0.35	0.41
MIP-3β	0.11	0.24	−0.27	−0.14	−0.01	0.11	**−0.69** ^**^
MIP-1α	−0.23	0.41	0.23	0.16	0.32	−0.12	0.34
GRO β	−0.07	**0.54** ^**^	−0.29	−0.06	**0.50** ^**^	0.22	−0.08
PD-L1	0.12	0.36	0.19	0.16	**0.55** ^**^	−0.07	0.31
CD40 L	0.42	0.10	−0.09	0.01	−0.00	−0.00	0.10
IFN-β	−0.35	0.19	0.03	0.01	−0.18	−0.25	−0.13
G-CSF	0.10	0.16	−0.09	0.15	**0.67** ^**^	−0.01	**0.46** ^*^
Granzyme B	−0.06	**0.48** ^*^	0.31	0.23	0.38	0.25	0.23
TRAIL	0.12	−0.07	0.13	0.14	0.38	0.01	0.34
EGF	0.32	0.19	0.32	−0.04	0.12	−0.00	0.23
PDGF-AA	−0.21	0.24	−0.36	−0.12	0.14	0.09	−0.04
PDGF-AB/BB	−0.28	0.25	−0.09	0.00	0.42	0.07	0.09
TGF-α	0.13	0.34	0.02	0.18	**0.48** ^*^	0.15	0.14
VEGF	−0.21	−0.01	−0.18	0.03	0.24	0.03	0.05
FGF β	−0.36	−0.02	0.02	0.17	0.01	−0.14	−0.15

Pearson correlation analysis evaluated possible links between aqueous inflammatory factors and visual acuity, and OCTA parameters. ^*^p < 0.05; ^**^p < 0.01. Bold indicating p value was statistically significant. CRT, central retinal thickness; FAZ, foveal avascular zone; PERIM, perimeter of fovea; ILM-IPL, inner limiting membrane-inner plexiform layer; SVD, superficial vessel density; and DVD, deep vessel density.

The plasma levels of IL-17E, IL-33, INF-β, GROβ, and FGFβ positively correlated with the FAZ. No correlation was observed between the plasma levels of inflammatory mediators and visual acuity, PERIM, ILM-IPL, SVD, and DVD (Table 5).

**Table 5:** Correlation between plasma inflammatory factors and BCVA and OCTA parameters in plasma levels of RVO patients (*n* = 19).

Variables	Log MARr	CRT	FAZ	PERIM	ILM-IPL	SVD	DVD
	*r*	*r*	*r*	*r*	*r*	*r*	*r*
IL-17E	0.26	−0.09	**0.49** ^*^	0.15	−0.12	0.15	0.17
Flt-3 L	−0.18	−0.03	0.36	0.36	−0.17	−0.23	−0.10
IL-3	0.04	−0.01	0.34	−0.11	−0.14	−0.03	0.15
IL-8	−0.08	−0.20	−0.02	−0.08	−0.10	0.36	0.01
IL-33	0.32	−0.07	**0.50** ^*^	0.15	−0.11	0.12	0.14
MIP-3β	0.10	−0.17	0.12	0.36	0.02	0.18	−0.11
MIP-1α	0.20	0.23	0.26	0.04	0.10	0.35	0.06
GRO β	0.28	0.09	**0.50** ^*^	0.07	−0.03	0.13	0.17
PD-L1	0.13	0.18	0.22	0.07	0.00	0.05	−0.00
CD40 L	0.20	0.01	0.32	−0.08	−0.15	−0.01	0.13
IFN-β	0.41	−0.05	**0.47** ^*^	0.36	−0.10	0.13	0.05
G-CSF	0.29	−0.03	0.42	0.30	−0.05	0.11	0.13
Granzyme B	−0.14	0.20	0.41	−0.04	−0.14	−0.03	0.17
TRAIL	0. 31	0.00	0.32	0.11	−0.04	−0.04	0.05
EGF	0.26	0.01	0.37	0.10	−0.10	0.10	0.12
PDGF-AA	0.15	−0.14	0.37	0.15	−0.10	0.10	0.15
PDGF-AB/BB	−0.08	−0.04	0.37	0.26	−0.07	0.01	0.13
TGF-α	0.40	−0.10	0.40	0.12	−0.11	0.16	0.12
VEGF	0.20	0.06	0.43	0.33	−0.03	0.09	0.07
FGF β	0.37	−0.10	**0.49** ^*^	0.14	−0.10	0.14	0.12

Pearson correlation analysis evaluated possible links between plasma inflammatory factors and visual acuity, OCTA parameters. ^*^p < 0.05; ^**^p < 0.01. Bold indicating *p* value was statistically significant. CRT, central retinal thickness; FAZ, foveal avascular zone; PERIM, perimeter of fovea; ILM-IPL, inner limiting membrane-inner plexiform layer; SVD, superficial vessel density; and DVD, deep vessel density.

We also found a positive correlation between blood GLU and retinal PERIM. The circulating levels of TC, HDLC, and LDLC positively correlated with FAZ. In addition, the level of APTT positively correlated with the CRT and the level of FIB positively correlated with the SVD (Table 6). Our results suggest a link between circulating levels of glucose, cholesterol, and coagulation factors and retinal structural alteration in RVO.

**Table 6:** Correlation between BCVA, OCTA parameters and blood test parameters in RVO patients (*n* = 20).

Variables	Log MAR	CRT	FAZ	PERIM	ILM-IPL	SVD	DVD
	*r*	*r*	*r*	*r*	*r*	*r*	*r*
GLU	0.19	−0.18	0.22	**0.45** ^*^	−0.05	0.28	−0.12
TG	−0.11	0.08	0.30	0.44	0.00	0.42	0.09
TC	−0.15	−0.03	**0.52** ^*^	0.36	0.10	0.22	0.15
HDLC	−0.26	−0.08	**0.53** ^*^	0.07	−0.03	0.02	0.11
LDLC	−0.11	−0.03	**0.52** ^*^	0.27	0.04	0.14	0.13
PT	0.15	0.19	−0.27	−0.20	0.07	−0.14	0.03
PTINR	0.17	0.20	−0.26	−0.19	0.09	−0.12	0.03
TT	0.38	−0.16	−0.14	0.03	0.21	0.01	−0.02
APTT	0.06	**0.46** ^*^	0.03	−0.06	0.26	0.07	−0.09
FIB	0.12	−0.02	−0.04	0.20	0.02	**0.47** ^*^	0.19

Pearson correlation analysis evaluated possible links between blood test and visual acuity, OCTA parameters. ^*^p < 0.05; ^**^p < 0.01. Bold indicating *p* value was statistically significant. CRT, central retinal thickness; FAZ, foveal avascular zone; PERIM, perimeter of fovea; ILM-IPL, inner limiting membrane-inner plexiform layer; SVD, superficial vessel density; DVD, deep vessel density. GLU, glucose; TG, triglyceride; TC, total cholesterol; HDLC, high-density lipoprotein cholesterol; LDLC, low density lipoprotein cholesterol; PT, prothrombin time; PTINR, prothrombin time international normalized ratio; TT, thrombin time; APTT, activated partial thromboplastin time; and FIB, fibrinogen.

### 3.5. Correlation between plasma and aqueous humor levels of inflammatory mediators

**Figure 1:**
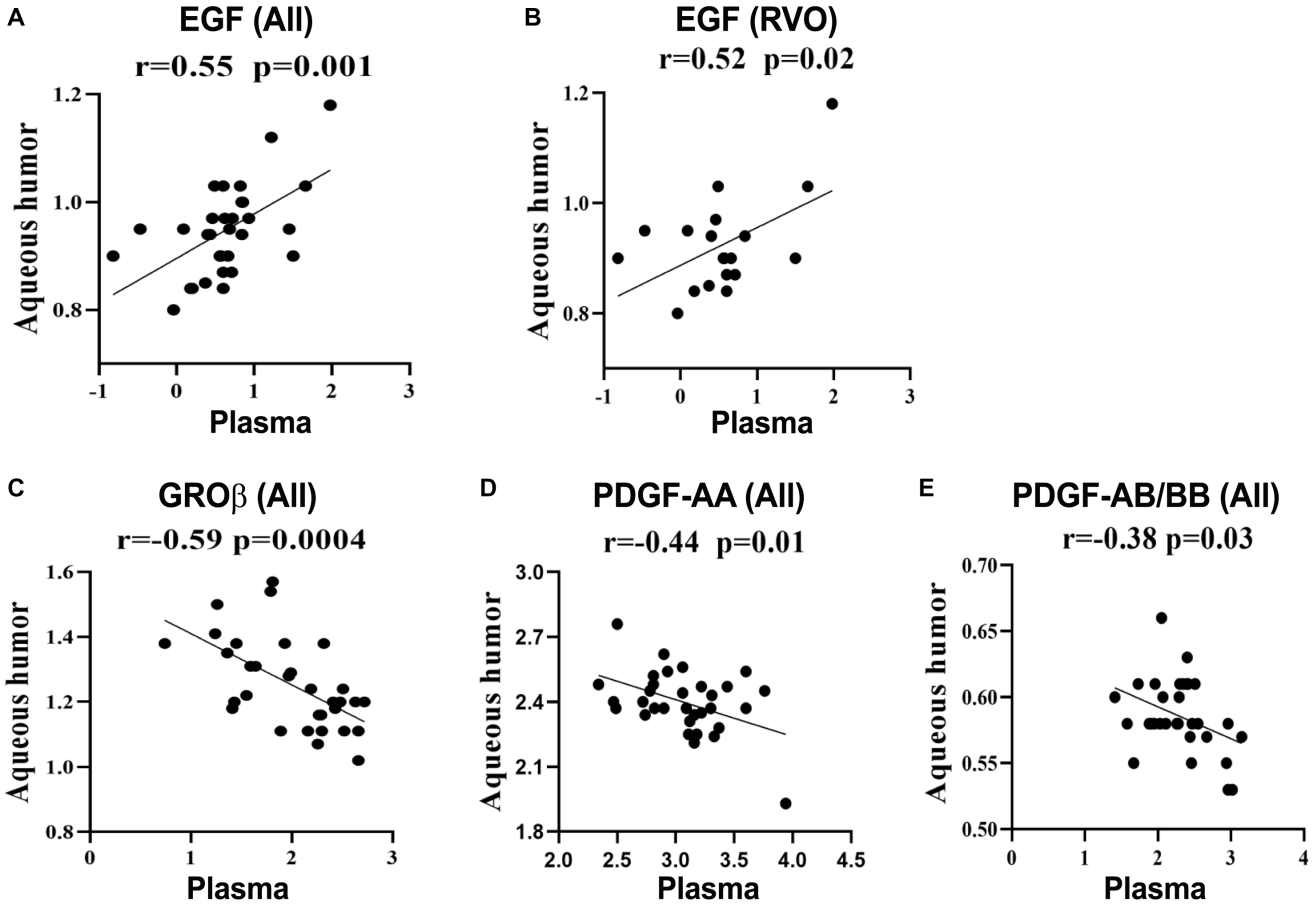
Correlations between the plasma and aqueous humor levels of immune mediators. Data were log-transformed and correlations were calculated as Pearson’s correlation coefficient (*r*). **(A,C–E)** Correlation analysis of GROβ, EGF, PDGF-AA, and PDGF-AB/BB in RVO patients and cataracts. **(B)** Correlation analysis of EGF in RVO patients.

The plasma level of EGF positively correlated with that in aqueous humor in all participants (Figure 1A), but the correlation became less significant in RVO patients (Figure 1). The plasma levels of GROβ, PDGF-AA, and PDGF-AB/BB negatively correlated with those in aqueous humor. No correlation was detected in other inflammatory mediators between the plasma and aqueous humor. Our results suggest that the majority of intraocular inflammatory mediators are independent of their counterparts in blood circulation.

### 3.6. PPI and enrichment analysis of measured inflammatory factors

**Figure 2:**
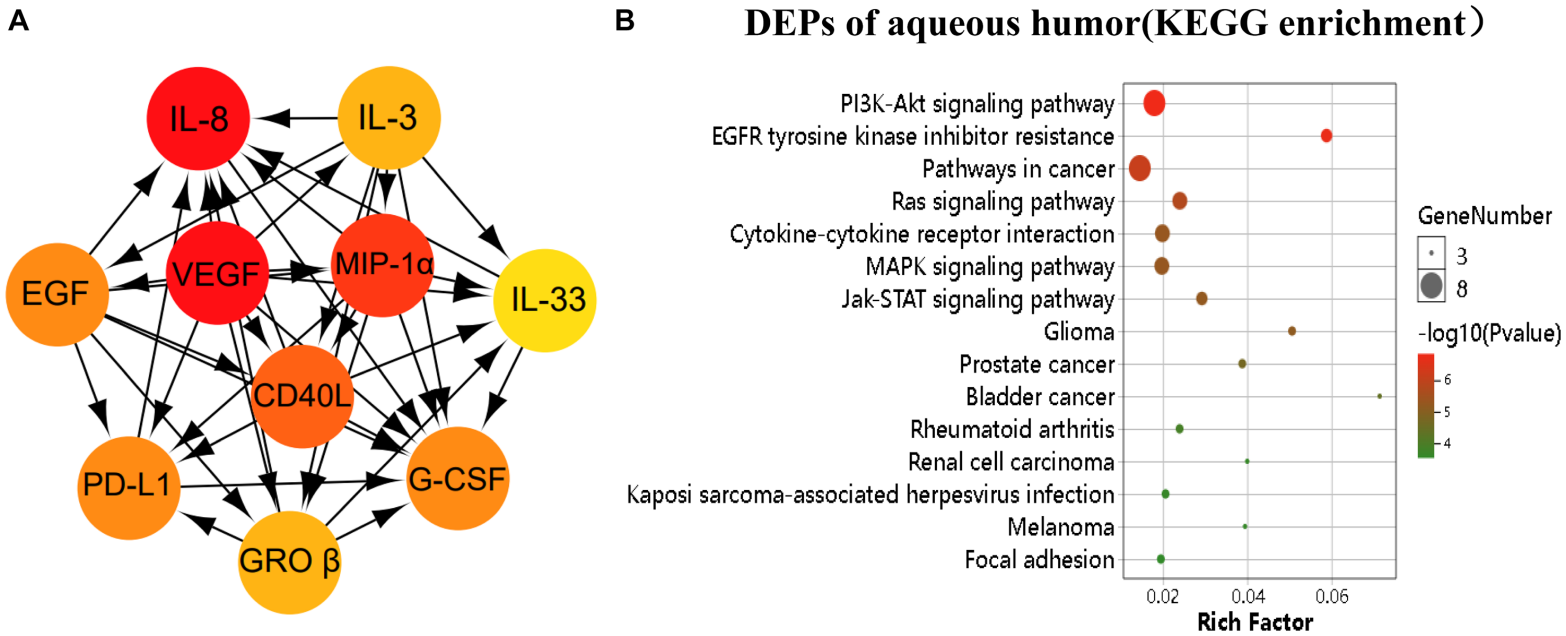
Pathway analysis of the differentially expressed proteins (DEPs). The DEPs were identified as *p* < 0.05. **(A)** The image showed the top 10 highest degree of aqueous connection DEPs from Cytohubba. Arrows indicate the direction of protein–protein interaction. The darker the color, the higher degree of connectivity of the nodes. **(B)** The TOP 15 aqueous DEPs enriched pathways from KEGG analysis.

To understand the pathways associated with RVO, we constructed a PPI network using the STRING database with the 17 differentially expressed proteins (DEPs) uncovered in our analysis. The analysis obtained 17 nodes and 78 edges with a confidence score > 0.4. Figure 2A showed the top 10 highest degree connection factors, namely, IL-8, VEGF, MIP-1α GROβ, CD40L, G-CSF, PD-L1, EGF, IL-3, and IL-33. VEGF appears to be the upstream protein that can affect all other proteins, whereas IL-8, G-CSF, and IL-33 are the downstream proteins affected by others in the PPI network (Figure 2A). KEGG analysis identified PI3K-Akt, EGFR tyrosine kinase inhibitor resistance, cancer, Ras, cytokine-cytokine receptor interaction, MAPK, Jak/STAT, glioma, prostate cancer, and bladder cancer as significantly enriched pathways (Figure 2B).

## 4. Discussion

In this study, we show that RVO patients had significantly higher intraocular levels of inflammatory mediators compared with senile cataract controls. Five (Flt-3 L, IL-8, MIP-3β, GROβ, and VEGF) out of 20 inflammatory mediators in the aqueous humor were significantly higher in RVO patients. The changes in intraocular cytokines were more pronounced in CRVO than in BRVO. Eleven out of 20 cytokines were significantly higher in CRVO than in BROV. KEGG enrichment analysis showed that the PI3K-Akt, Ras, MAPK, and Jak–STAT signaling pathways were affected by these cytokines. Importantly, we found that the alterations in intraocular cytokines were independent of systemic inflammation as none of the inflammatory cytokines was significantly increased in the plasma in RVO patients. Our results suggest that retinal pathologies in RVO may be driven primarily by dysregulation of intraocular immune response but not systemic immune mediators.

The RVO is due to abnormal thrombosis, which can arise from abnormal endothelial cell activation, misbehavior of circulating immune cells and platelets activation. The plasma IL-8, IL-17E, MIP-1α, MIP-3β, G-CSF, and IFNβ are most likely produced by circulating immune cells, and PDGF-AA and PDGF-AB/BB are known to be released by active platelets. The fact that none of them was altered in RVO patients suggests that abnormal behavior of circulating immune cells and platelets is unlikely the cause of thrombosis. Surprisingly, the plasma level of GROβ was significantly lower in the RVO patients, particularly in those with less than 1-month disease duration (seven times lower than controls), suggesting a likely protective role of circulating GROβ in RVO. Interestingly, we found that the plasma levels of IL-17E, EGF, IL-33, MIP-1α, TGF-α, and FGFβ positively correlated with disease duration, indicating that these circulating factors may be an active response to RVO-mediated retinal ischemic injury.

We found that RVO (particularly CRVO) patients, had significantly higher intraocular levels of inflammatory cytokines/mediators including Flt-3 L, IL-8, IL-33, MIP-1α, RGOβ, G-CSF, PDGF-AA, PDGF-AB/BB, TGF-α, and VEGF. The higher intraocular inflammation may lead to retinal vascular endothelial activation, which may trigger abnormal thrombosis. The crosstalk between inflammation and coagulation and their involvement in thrombotic diseases is well recognized (Foley and Conway, 2016). For example, G-CSF and IL-8 can induce neutrophil extracellular traps (NETs), which can enhance coagulation (Demers et al., 2012; Alfaro et al., 2016; Thålin et al., 2019). TNF-α can facilitate the crosstalk between inflammation and thrombosis by triggering the NF-κB pathway (Muralidharan-Chari et al., 2016). VEGF can induce tissue factors in endothelial cells and tumor cells, activating coagulation and fibrin formation (Salgado et al., 2002). Although higher levels of intraocular inflammation can be explained by RVO-mediated retinal damage, it is possible that these eyes may also have higher basal levels of inflammatory cytokines, which may stimulate retinal vascular endothelial cells and contribute to the development of RVO.

We found positive correlations between parameters of macular oedema (i.e., CRT and ILM-IPL) and intraocular inflammatory mediators including GROβ, Granzyme B, Flt-3 L, IL-33, PD-L1, and G-CSF, suggesting that they may contribute to retinal vascular leakage, fluid accumulation, and neuronal damage in RVO. The aqueous level of MIP-3β negatively, but G-CSF positively correlated with DVD, an indicator of deep layer retinal vascular degeneration. G-CSF is an essential growth factor for microglia (Chitu et al., 2021). Retinal microglia in the inner retina are known to rely on IL-34 (O'Koren et al., 2019), whereas the microglia in the outer retinal layer are IL-34-independent, and they may rely on G-CSF. Microglia is an important component of neurovascular unit (Liu et al., 2020). Our results suggest that G-CSF may be protective, while MIP-3β may be detrimental to retinal microvasculature in RVO. Intravitreal injection of VEGF inhibitors is the standard of care for RVO-mediated macular oedema (Campochiaro et al., 2014a,b; Thach et al., 2014). Surprisingly, we did not detect any direct correlation between intraocular levels of VEGF and RVO-related macular changes. PPI analysis showed that VEGF is the upstream factor that can affect many other cytokines, including IL-8, IL-33, G-CSF, and GROβ (Figure 2A). Collectively, these cytokines can affect the PI3K-Akt, Ras, MAPK, and Jak–STAT signaling pathways (Figure 2B). The role of PI3K-Akt (Jacot and Sherris, 2011; Ma et al., 2022), Ras (Wang et al., 2015), MAPK (Supanji et al., 2013; Moustardas et al., 2023), and Jak–STAT (Chen et al., 2016, 2019; Hombrebueno et al., 2020; Cho et al., 2022) pathways in inflammatory and degeneration retinal diseases such as diabetic retinopathy and age-related macular degeneration has been well appreciated. Our results suggest that they may also be involved in RVO-mediated retinopathy.

Interestingly, we found positive correlations between FAZ and systemic factors, including the plasma levels of IL-17E, IL-33, GROβ, IFN-β, FGF β, and circulating lipid/cholesterols (TC, HDLC, and LDLC). FAZ is the most sensitive central area of the macula, and changes in its shape and size can pose great threats to vision. Progressive and irregular expansion of FAZ has been observed in RVO eyes (Fan et al., 2022), and changes in FAZ are related to capillary remodeling in the macular area (Tripathy et al., 2021). Our results suggest that circulating factors may be involved in retinal microvascular remodeling in RVO.

The strengths of the study include (1) the simultaneous measurement of inflammatory mediators in the blood and aqueous humor from the same participants; (2) comprehensive clinical and laboratory evaluations of the participants. The study has several limitations. First, the number of participants enrolled in this study was relatively small (e.g., six CRVO and 14 BRVO). Second, the OCTA parameters (e.g., FAZ, PERIM, and ILM-IPL) were only conducted in RVO patients but not in control cataract patients. Third, the study was conducted in a single center and the results can only reflect the biological feature of RVO in the local ethical population. Replication of the findings with a larger sample size and in multiple ethnic groups is necessary to confirm our results. However, it should be noted that single center study reduces procedure-related variation and increases the reliability of the results in small sample size studies.

In conclusion, we show that intraocular inflammation in RVO patients is driven primarily by local factors but not circulating immune mediators. Intraocular inflammation may be involved in the development of RVO and contribute to macular oedema through the PI3K-Akt, Ras, MAPK, and Jak/STAT signaling pathways. The blood levels of cholesterols and the levels of IL-17E, IL33, RGOβ, and FGF-b may affect retinal microvascular remodeling in RVO.

## Data availability statement

The original contributions presented in the study are included in the article/Supplementary material, further inquiries can be directed to the corresponding authors.

## Ethics statement

The studies involving human participants were reviewed and approved by Institutional Review Board (IRB) of the Changsha Aier Eye Hospital. The patients/participants provided their written informed consent to participate in this study.

## Author contributions

HX and ZC conceived and designed the study. YZ, HL, JC, and HT acquired data. JQ, WP, and YZ analyzed the results. HX, ZC, JQ, and YZ discussed and interpreted the data. JQ, YZ, and HX wrote the manuscript, and YZ, JQ, HL, SL, TG, JC, WP, HT, JW, HX, and ZC reviewed the manuscript. All authors contributed to the article and approved the submitted version.

## Funding

This research was supported by Research and Development Plan of Key Fields in Hunan Province (2020SKC2007), the Natural Science Foundation of Hunan Province (2021JJ30046), the Science Research Fund of AIER Eye Hospital Group (AF2201D12, AM2001D4, AM1913D1, and AR2003D1), Hunan Province Optometry Engineering and Technology Research Center, and Hunan Province International Cooperation Base for Optometry Science and Technology.

## Conflict of interest

The authors declare that the research was conducted in the absence of any commercial or financial relationships that could be construed as a potential conflict of interest.

## Publisher’s note

All claims expressed in this article are solely those of the authors and do not necessarily represent those of their affiliated organizations, or those of the publisher, the editors and the reviewers. Any product that may be evaluated in this article, or claim that may be made by its manufacturer, is not guaranteed or endorsed by the publisher.

## Footnotes

^1^ https://www.omicshare.com/tools
