# Supplementary material for: Increased intraocular inflammation in retinal vein occlusion is independent of circulating immune mediators and is involved in retinal oedema
Source: Front Neurosci. 2023 Jul 24;17:1186025. doi: 10.3389/fnins.2023.1186025 (PMC10405077; doi:10.3389/fnins.2023.1186025)
Supplement: Supplementary file 1 [file Table_1.docx]

Supplementary Material

**Increased intraocular inflammation in retinal vein occlusion is independent of circulating immune mediators and is involved in retinal oedema**

Yufan Zhou and Jinyan Qi contributed equally to the work and share the first authorship

* Correspondence: Heping Xu <heping.xu@qub.ac.uk> and Zhongping Chen <chenzhongping@aierchina.com>

**Table S1**. Disease duration and inflammatory factors in plasma RVO patients.

|  | **Aqueous humour** | | |  | **Plasma** | | |
| --- | --- | --- | --- | --- | --- | --- | --- |
| **Variables** | **≤1m**  **n=10** | **＞1m**  **n=9** | **r** |  | **≤1m**  **n=10** | **＞1m**  **n=9** | **r** |
| IL-17E | 20.42±2.62 | 22.29±2.34 | 0.13 |  | 5.00±2.31 | **10.60±8.90*^, a^** | **0.45*^, b^** |
| Flt-3 L | 39.88±9.03 | 42.14±14.48 | 0.85 |  | 59.76±29.01 | 69.24±29.34 | 0.18 |
| IL-3 | 24.11±1.36 | 24.42±3.10 | 0.34 |  | 2.18±1.99 | 8.50±15.19 | 0.34 |
| IL-8 | 112.19±83.05 | 89.41±85.43 | 0.25 |  | 1.40±0.80 | **7.83±10.90*^, a^** | **0.54*^, b^** |
| IL-33 | 17.90±0.93 | 18.02±1.64 | 0.89 |  | 6.30±3.02 | **13.20±11.78*^, a^** | **0.46*^, b^** |
| MIP-3β | 14.22±10.78 | 12.05±6.32 | 0.74 |  | 76.09±26.73 | 83.63±41.54 | 0.08 |
| MIP-1α | 13.46±1.47 | 13.09±1.61 | 0.58 |  | 10.32±6.25 | **21.61±10.88*^, a^** | **0.51*^, b^** |
| GRO β | 22.33±6.50 | 20.88±8.07 | 0.54 |  | 45.25±28.04 | 119.50±135.01 | 0.35 |
| PD-L1 | 102.52±9.37 | 105.91±16.14 | 0.63 |  | 92.65±127.98 | 144.09±113.21 | 0.28 |
| CD40L | 1316.33±101.98 | 1359.83±158.51 | 0.53 |  | 216.26±166.56 | 909.44±1625.33 | 0.43 |
| IFN-β | 4.09±0.41 | 3.80±0.36 | 0.13 |  | 2.29±2.53 | 4.53±4.90 | 0.39 |
| G-CSF | 51.05±128.13 | 72.28±181.64 | 0.82 |  | 8.23±5.38 | 15.54±14.40 | 0.32 |
| Granzyme B | 8.82±1.97 | 10.47±4.18 | 0.31 |  | 1.46±2.04 | 7.15±12.04 | 0.28 |
| TRAIL | 26.27±1.87 | 26.28±1.27 | 0.95 |  | 46.00±15.98 | 61.45±42.41 | 0.21 |
| EGF | 8.04±0.95 | 9.16±2.60 | 0.26 |  | 2.54±1.80 | 20.42±29.98 | **0.55*^, b^** |
| PDGF-AA | 335.39±103.37 | 245.77±79.40 | 0.05 |  | 995.74±1109.40 | 2386.12±2709.09 | 0.42 |
| PDGF-AB/BB | 3.81±0.22 | 3.90±0.31 | 0.55 |  | 226.20±254.22 | 373.46±465.95 | 0.21 |
| TGF-α | 10.00±0.77 | 9.98±0.87 | 0.94 |  | 5.13±3.69 | **13.06±11.92*^, a^** | **0.52*^, b^** |
| VEGF | 810.15±946.41 | 850.98±885.34 | 0.87 |  | 43.71±19.41 | 83.08±69.75 | 0.48 |
| FGFβ | 12.84±1.77 | 12.85±3.36 | 0.84 |  | 5.53±3.64 | **17.04±17.62*^, a^** | **0.53*^, b^** |

^a^, Multivariable linear regression analysis of inflammatory factors between patients with ≤ 1 month disease duration and those > 1 month disease duration after adjusting for BMI and age. ^b^, Pearson correlation analysis the link between aqueous humour levels of inflammatory factors and disease duration. * p<0.05.

**
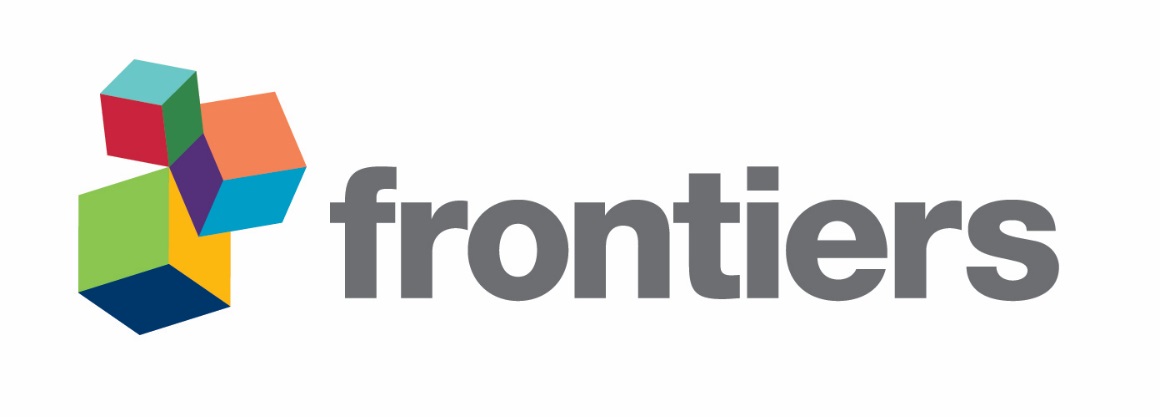
**

.
